# The Winter Drive of the Medical Forces with the American Army in France

**Published:** 1918-11

**Authors:** 


### THE WINTER DRIVE OF THE MEDICAL FORCES WITH THE AMERICAN ARMY IN FRANCE

The Surgeon-General, the Chief Surgeon, the Chief Consultant in Medicine, the Head of the Division of Laboratories and Infectious Diseases, the Head of the Sick and Wounded Office in the Chief Surgeon's Office, and other high officials in the Medical Department of the A. E. F., have not forgotten last winter's experience with the acute respiratory infections which caused the death of so many soldiers in the training camps of the United States Army. They have realized that, during the coming winter, diseases which thrive in cold weather, when men are crowded together in badly ventilated rooms, are greater enemies to American soldiers than German submarines, Krupp guns, and Boche air-bombs, all combined; and they have planned months ahead for the present campaign that is being waged against acute respiratory infections.

The opening guns of the campaign were fired at the September meeting of the Research Society, when a session was devoted to the discussion of the " Acute Respiratory Infections ". At this meeting, more than two hundred medical officers had the privilege of hearing the experience of the French and British Armies with respiratory diseases during the past four winters; and they were also informed by a representative from the Chief-Surgeon's Office that, in our first year of the war, acute respiratory infections caused more deaths and a higher non-effective rate than any other group of diseases that had occurred among the soldiers of the A. E. F. Plans for a winter campaign against these diseases were also discussed.

The facts brought out at this meeting regarding acute respiratory infections were published in the October number of *War Medicine*, and a copy has been sent to every medical officer in the A. E. F.

### The October Meeting of the Research Society.

There was not time at the September meeting of the Research Society for a full discussion of the very important part played by the streptococcus haemolyticus in the pneumonias and empyemas that caused many deaths among our soldiers last year. For this reason, one session of the October meeting was given over to the continuation of the discussion on " Streptococcus Infections of the Respiratory Tract ". On this occasion the British experience with streptococcus infections was presented, and prominent American clinicians, who are now in France, gave accounts of the investigations, with the conclusions drawn therefrom, that were carried out during the epidemics of pneumonia in army camps in the United States. They brought out the fact that the streptococcus haemolyticus, so dreaded by surgeons, was the organism that had caused the greatest number of cases of pneumonia and empyema following measles and influenza, and that it also was the etiological factor in many of the cases of primary pneumonia with irregular courses.

These medical officers also outlined measures for handling the acute respiratory diseases that have been found practical in army hospitals. The complete isolation of respiratory cases in separate hospitals or pavilions was advocated; but in time of epidemics, when such patients have to go to general hospitals, the system of isolation in " cubicles " was advised, although care should be taken that they be so arranged that patients may have sufficient air-space. The need for an " aseptic medical technic " in handling respiratory infections was emphasized. Plenty of fresh air, both in the treatment and in the prevention of respiratory infections, was stressed by some of the speakers as being most important. The discussions on streptococcus infections of the respiratory tract at the October meeting of the Research Society are published in this number of *War Medicine*, and the information contained in them is therefore available for medical officers.

### The Medical Department's Fight.

For several months the Chief Surgeon's Office has been bombarding the enemies responsible for the heavy losses we have had from acute respiratory infections by sending out in the Weekly Bulletins a lot of valuable information on influenza and pneumonia, with practical instructions for the measures to be employed in preventing those diseases among soldiers. Extracts from these bulletins are published in this number of *War Medicine*, and this material should be utilized to the fullest extent by medical officers in making the fight in the present drive against winter diseases.

The Division of Laboratories and Infectious Diseases of the A. E. F. is also making a strong counter-attack on the respiratory infections that have already begun their big winter offensive. Representatives from this Division are everywhere in the A. E. F., studying conditions and instructing medical officers as to their part in the fight. The American Red Cross in France recently published for this Division " A Bulletin on Infectious Diseases ", and the parts dealing with prevention of acute respiratory infections are so practical that, for the information of medical officers who may not receive copies, they are reproduced in *War Medicine.*

### A Text Book on Acute Respiratory Infections.

Believing that " medical journals are the doctors' best text books ", the Bureau of Medical Publications of the American Red Cross is endeavoring in this number of *War Medicine* to provide medical officers with an up-to-date text book on the etiology and prevention of the acute respiratory infections. We believe that no text book in existence presents a more practical treatise on the hygiene of respiratory infections, as adapted to military needs, than the material which has been gathered from many sources and published in this issue of *War Medicine.* Medical officers in France, whether they are engaged in general work or in any of the medical and surgical specialities, should study this text book as they have never studied one before, until they know every phase of the causes of, and the methods for preventing, the acute respiratory diseases.

This winter's drive against the acute respiratory infections is perhaps the most important one in which the A. E. F. will be engaged, and medical officers must lead the fight everywhere. That medical officer is a " slacker " who does not learn all that he can about the diseases which are causing the greatest losses to our armies, and who does not put forth every ounce of energy he possesses in a work which has for its aim the saving of the lives of our soldiers who have been drafted into this war to fight the battles of freedom and democracy. It is a fight in which each medical officer in France should do his part; and with united, systematic and thorough work, thousands of lives may be saved, and tens of thousands of fighting men kept fit for service at the front, who, without heroic effort on our part, will become infected with influenza and pneumonia and fill our hospitals, thus giving the American Expeditionary Forces high non-effective and death rates that will reflect discredit upon its Medical Department.

